# Residual Activity of Methoprene and Novaluron as Surface Treatments to Manage the Flour Beetles, *Tribolium castaneum* and *Tribolium confusum*

**DOI:** 10.1673/031.012.9501

**Published:** 2012-08-12

**Authors:** Frank H. Arthur, Emily A. Fontenot

**Affiliations:** USDA-Agricultural Research Service-Center for Grain and Animal Health Research, 1515 College Avenue, Manhattan, Kansas, USA; ^§^Current Address: Insect Pest Control Laboratory, Joint FAO/IAEA Division of Nuclear Techniques in Food and Agriculture, IAEA, Wagramerstrasse 5, Vienna 1400, Austria

**Keywords:** efficacy, insecticides, surfaces

## Abstract

The juvenile hormone analog methoprene, and the chitin synthesis inhibitor novaluron, were evaluated by exposing late-stage larvae of *Tribolium castaneum* (Herbst) or *Tribolium confusum* (Jacqueline DuVal) (Coleoptera: Tenebrionidae) to it. The larvae were exposed to it in food material, on concrete, on plywood, and on floor tile. Larvae of *T. castaneum* were more susceptible than *T. confusum* larvae to both methoprene and novaluron on all surfaces. A further evaluation was done by exposing adult *T. confusum* to methoprene and novaluron through food placed on concrete treated with methoprene and novaluron, and then assessing resulting progeny production. The emergence of adults with normal morphology was reduced for both chemicals, with more malformed adults appearing in the methoprene treatment, and fewer adults of any form emerging in the novaluron treatment. The results show direct exposures to larvae, or determining progeny production from exposed adults, are valid methods for assessing the susceptibility of flour beetles to insecticides.

## Introduction

Insect growth regulators (IGRs) are effective against stored product insects (Oberlander et al. 1997; Oberlander and Silhacek 2000). Various methods have been used in research studies to evaluate effectiveness of IGRs, including incorporating the IGR into the diet of the insects, with the usual result being almost complete inhibition of adult emergence, depending on the target species, specific IGR, and dosage rate (Oberlander et al. 1997). Evaluation of an IGR through exposure in treated diet may not accurately reflect exposure in field conditions, where immature and adult insects encounter food sources that may be deposited on treated surfaces.

The IGRs hydroprene, methoprene, and pyriproxyfen are juvenile hormone analogs registered for general surface treatments in the USA to control stored product insects, including the red flour beetle, *Tribolium castaneum* Herbst, and the confused flour beetle, *Tribolium confusum* Jacquelin du Val (Coleoptera: Tenebrionidae). Hydroprene is volatile, and does not have residual efficacy on a treated surface (Arthur and Hoernemann 2004). Pyriproxyfen has residual efficacy as a surface treatment, though there is some variation depending on the particular treated surface (Arthur et al. 2009). Methoprene is persistent as an aerosol application (Arthur 2010), and surface treatments could be effective as well, because methoprene is labeled as a grain protectant in the USA (Arthur 2004) and Australia (Daglish and Wallbank 2005; Daglish 2008).

Novaluron is a relatively new chitin synthesis inhibitor that is under development for agricultural and fruit pest species in the USA (Hoffman et al. 2009; Kamminga et al. 2009; Beauzulin et al. 2010; Wise et al. 2010). The only published studies in which novaluron was evaluated against stored product insects are by Kostyukovsky and Trostanetsky (2006, 2008), who reported suppressed development of *T. castaneum* when exposed on flour treated with novaluron. The objectives of this study were to evaluate methoprene and novaluron against *T. castaneum* and *T confusum* by exposing late-stage larvae on treated surfaces, and to assess efficacy through the exposure of *Tribolium* adults, and the assessment of eventual progeny production.

## Materials and Methods

### Methoprene on Different Surfaces

Exposure arenas consisting of concrete, plywood, and tile were constructed using the bottom portion of a plastic Petri dish that had an approximate measured area of 62 cm^2^. A driveway patching material (Rockite®, http://www.wagnercompanies.com/rockite.aspx) purchased locally was used to create a concrete surface. Approximately 3.2 kg of the dry powder was mixed with 1.6 L of tap water to create a slurry, and the 120 Petri dishes (hereby termed “arenas”) were filled to a depth of about 0.5 cm with the slurry. The patching material was allowed to dry for two days. Wood arenas were created by cutting 48 circular discs from plywood that was 1.25 cm thick. A disc was placed into each of the arenas described above, and the circumference was caulked. One hundred and twenty tile arenas were created by making a wood surface, cutting a piece of self-adhesive polyurethane tile to fit the wood disc, gluing the tile onto the wood, and then caulking the circumference, as described for the wood surface.

The formulation of methoprene used in this test was Diacon II® (ww.diacon2.com), which is a 33.6% active ingredient (AI) emulsifiable concentrate (EC), 288 mg AI/mL. Label directions specify 1 mL of the EC in 3.8 L to cover 94 m^2^, which gives an approximate rate of 3.0 mg AI/m^2^. Four replicate insecticide solutions were formulated by mixing 0.132 mL of the EC into 500 mL of distilled water. Each replicate solution was used to treat 24 of the arenas of each surface at the label volume rate of approximately 40 mL of formulated spray/m^2^, which required approximately 0.25 mL of spray for each arena. Spray solutions were applied using a Badger 100 artists’ airbrush (www.badgerairbrush.com). A separate replicate of 24 arenas of each surface served as the untreated control by spraying each arena with distilled water at the rate of 0.25 mL per arena, using a different airbrush than the one used for the treatments.

The process of exposing *T. castaneum* and *T. confusum* on the treated surfaces at each of the exposure intervals was as follows. Both species were from pesticide susceptible laboratory cultures at the Center for Grain and Animal Health Research (CGAHR), reared on a diet of 95% unbleached whole-wheat flour, and 5% brewer's yeast. All of these cultures were maintained at 27° C and 60% relative humidity, in constant darkness. These cultures had been maintained for approximately 20 years. For each surface, exposure time, and insect species, there were the four replicates of treated arenas, as described above, and the untreated control (two sets for each species). On one set of four replicates and the untreated control for a given surface, ten 4-week-old late instars of *T. castaneum* were placed in each arena, along with approximately 500 mg of the rearing media. The same procedure was done for exposure of *T. confusum.* After the arenas were set up, the lids were placed on them, and all arenas were set inside an incubator with the same environmental conditions as used for the laboratory cultures.

After three weeks, the arenas were removed from the incubator, and the adults that were considered to be normal in morphological appearance with no visible defects (hereafter termed adult emergence) were counted in all treated replicates, and in the controls. At this time, emergence was generally complete in the controls, so the number of emerged adults was recorded, and the arenas were discarded. The number of emerged adults in the arenas treated with methoprene was also recorded, but these arenas were put back into the incubator for another three weeks as a further check on adult emergence. After three weeks, the arenas were examined again, but there was never any further adult emergence on any surface for any of the bioassays beyond what was originally recorded. Hence, the variable analyzed in the test was the percentage of adult emergence. The General Linear Models (GLM) Procedure of the Statistical Analysis System (SAS Institute 2007) was used to analyze the experiment with bioassay month, species, and surface as main effects. Means for treatments were separated using the Waller-Duncan *k-ratio-t* test in the GLM Procedure, using the square root transformation, but actual means are reported.

### Novaluron on Concrete

Based on the results for Experiment 1, which suggested that survival was greater on concrete compared to wood and tile, a new test was initiated in which novaluron was evaluated for control of *T. castaneum* and *T. confusum,* using only the concrete arena surface. Technical novaluron dry powder, 97.7% purity, was obtained from BASF Corporation (www.basf.com). The label rate for the IGR hydroprene is approximately 20 mg AI/m^2^, while the label rate for methoprene is 3 mg AI/m^2^. Because of the difference in label rates between the two commercial IGRs, and the lack of data regarding efficacy of novaluron, an initial test was done by formulating the novaluron technical material to give application rates of 10, 20, and 30 mg AI/m^2^ as a comparison to the label rate for hydroprene. Each of the three concentrations was formulated by weighing 9.6, 19.2, and 28.2 mg of technical novaluron into 25 mL of water to produce the concentrations of 10, 20, and 30 mg AI/m^2^, respectively. There were four replicates of each concentration, and an untreated control was included in the series with the three concentrations.

Forty concrete arenas were created as described above. There were five replicates for each treatment series (three concentrations plus the untreated control). For each replicate and concentration, two concrete arenas were sprayed with 0.25 mL of formulated spray using the artists’ airbrush. Ten 4-week-old *Tribolium castaneum* larvae were placed on one of the two arenas, and ten 4-week-old *T. confusum* larvae were placed on the other arena, along with 500 mg of flour. The arenas were held in the incubator, and assessed for adult emergence as described for Experiment 1, with the exception that the same arenas were used for the 0 and 2-month bioassays. The test was analyzed with bioassay time as a repeated measure, and species and concentration as the main effects, using the GLM Procedure in SAS. Data were transformed and analyzed as described above. Because there were only three concentrations, plus the untreated controls, regression analysis was not done, and the ordered sequencing was treated as a class variable.

### Entire Life-Stage Exposure

In this test, a different testing methodology was employed to evaluate novaluron in comparison to methoprene. Because of the lower application rate of methoprene compared to hydroprene, and the difficulty of formulating the technical novaluron at a low rate for comparison to methoprene, a commercial formulation of novaluron was used instead of the technical. This was Rimon Supra, 97.7 mg AI/ml EC, obtained from BASF and formulated to give solutions of 1.5, 3.0, and 4.5 mg AI/m^2^, which required 0.21, 0.42, and 0.81 mL, respectively, of Rimon Supra into 500 mL distilled water. The methoprene EC described in Experiment 1 was formulated as described to give a concentration of 3.0 mg AI/m^2^.

For this experiment, a different testing methodology was employed using concrete arenas, with *T. confusum* as the test species. Eighty arenas were created, 20 for each of four replicates. For each replicate, there were four residual bioassay times, 0, 2, 4, and 8 weeks post-treatment, conducted on separate arenas. For each set of replicates, the four insecticide concentrations (three for novaluron, one for methoprene) were formulated along with the untreated control. A series of four arenas was treated for each of the five treatments, as previously described. Three of the four arenas were held in the incubator for the residual bioassays, while the fourth one was held overnight for time 0 bioassays.

Bioassays were done as follows. At each testing time, 500 mg of flour was placed on each treated arena (or untreated control). Twenty mixed-sex adult beetles were placed in the arenas, which were then returned to the incubator. After one week, the adults were removed, and the arenas were put back into the incubator. After eight weeks, the arenas were removed, and emerged adults were classified as either morphologically normal or alive but deformed in some manner (twisted wings, unsclerotized integument). Adult-pupal intermediates, adults that failed to emerge from the puparium, or adults that died upon emergence, were not counted. This procedure was repeated at each of the residual bioassays. This test was analyzed as described previously using the GLM Procedure of SAS, with concentration and residual bioassays as the main effects, and total adult emergence, number of normal adults, and number of healthy adults as the variables of interest. Data were transformed as described above. Means were separated using the Waller-Duncan *k*ratio *t*-test. As the purpose of this test was to compare the two species at each of the concentrations, regressions were not done on weeks as an ordered sequence.

## Results

### Methoprene on different surfaces

One untreated control replicate was included each month, along with the four treatment replicates for each species. There was no difference in adult emergence on these untreated controls with respect to month or surface (*P* > 0.05 for the *F*-tests); therefore, no corrections for mortality were necessary.

Overall adult emergence in the controls was 59.2 ±8.4% for *T. castaneum* and 80.8 ±4.9% for *T. confusum.* In the methoprene treatments, main effects month, species, and surface were all significant at *P* < 0.01 (*F* = 41.1, df = 3, 72; F = 201.8, df = 1, 72; *F* = 40.1, df = 2, 72, respectively), and all interactions were also significant at *P* < 0.01. At all times except for week 0, more of the *T. confusum* larvae emerged as normal adults on all three surfaces compared to *T. castaneum,* indicating *T. confusum* was the more tolerant species (Table 1). At weeks 4, 8, and 16, the percentage of emerged adult *T. confusum* was usually greater on the concrete surface compared to the wood surface, indicating less efficacy of the methoprene on concrete compared to the other surfaces (Table 1).

### Novaluron on concrete

The main effects concentration and species, and the repeated measure time, were all significant at *P* < 0.01 (*F* = 18.2, df = 3, 27; *F* = 238.8, df = 1, 27; *F* = 5.9, df = 1, 22, respectively). The rate by time interaction was significant (*F* = 4.6, df = 3, 27, *P* = 0.011), but all other interactions were not significant (P ≥ 0.05). In the initial test at one day posttreatment, the 20 and 30 mg/m^2^ concentrations were effective against *T. castaneum* larvae, with 100% suppression of adult emergence at 30 mg/m^2^ (Table 2). In sharp contrast, none of treated with watera. the concentrations gave 100% suppression of *T. confusum* larvae, neither at this time nor at one month post-treatment (Table 1). In all comparisons of treatments at one day and at two months, adult emergence of exposed larvae was greater for *T. confusum* compared to *T. castaneum* (Table 2).

**Table 1.**
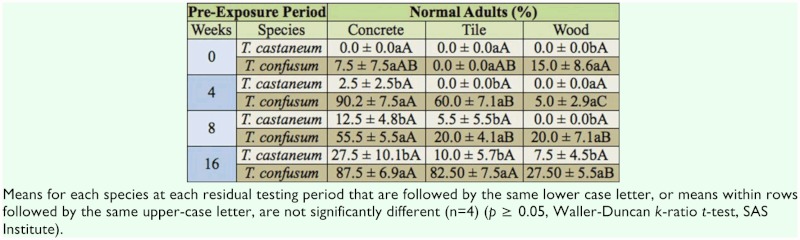
Percentage (mean ±SEM) of morphologically normal adults resulting from exposure of late instars of *Tribolium castaneum* and *Tribolium confusum* on concrete, tile, or wood treated with the methoprene at the rate of 3.0 mg active ingredient (A1)/m^2^. Bioassays were conducted | day after the surfaces were treated (week 0) and again after 4, 8 and I 6 weeks, using different treated arenas at each exposure interval.

**Table 2.**
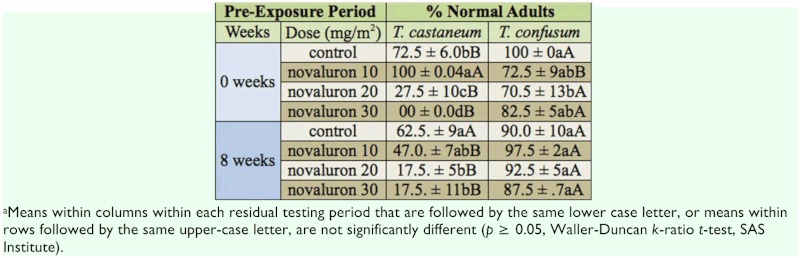
Percentage (mean ±SEM) of morphologically normal adults resulting from exposure of late instars of *Tribolium castaneum* and *Tribolium confusum* exposed on concrete treated with 10, 20, and 30 mg (A1)/m^2^ of novaluron. Bioassays were conducted 1 day after the concrete was treated and again 8 weeks later, using the same exposure arenas. Controls were^*a*^.

### Entire life-stage exposure

In this experiment, the main effect concentration was significant at *p*< 0.01 for all three variables of analysis, which were total adult progeny, number of deformed adults, and number of healthy adults (*F* = 78.8, *F* = 33.3, and *F* = 62.2, respectively; df = 4, 80). The residual bioassay time was not significant for total adult progeny or number of normal progeny (*F* = 2.1, *P* = 0.11; *F* = 1.0, *p* = 0.40; df = 3, 80), but was significant for the number of deformed adults (*F* = 4.9, df = 3, 80, *p* < 0.01). The interaction between the two main effects was significant at *p* < 0.01 for total adult progeny, number of deformed adults, and number of healthy adults (*F* = 3.0, *F* = 5.4, and *F* = 2.7; df = 12, 80). Total progeny production for all three rates of novaluron was always significantly lower than total progeny production in untreated controls, and in the methoprene treatment (Table 3). The number of deformed adult progeny was greater in the methoprene treatment than in the novaluron treatment; however, most of these deformed adults died upon emergence, or shortly thereafter. The number of living, healthy adults ranged between 0 and 6.2 in the novaluron treatments, and was usually lower than the corresponding number of normal adults in the methoprene treatment (Table 3).

**Table 3.**
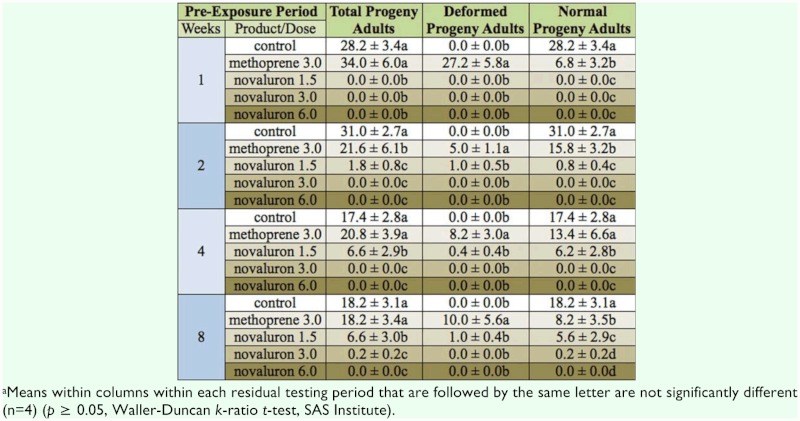
Number of total adult progeny, deformed progeny adults, and normal progeny adults (mean ±SE) resulting from exposure of larvae of *Tribolium confusum* on concrete surfaces treated with methoprene at the rate of 3.0 mg (A1)/m^2^ and novaluron applied at 1.5, 3.0, and 6.0 mg (A1) /m^2^. Controls were treated with water^*a*^.

## Discussion

*Tribolium castaneum* and *T. confusum* often show differences in susceptibility to contact insecticides used as surface treatments for adult control, depending on the specific insecticide (Arthur 2008b). Results from the portion of the study with exposures of 4-week-old larvae on surfaces treated with methoprene and novaluron showed a greater susceptibility of *T. castaneum* compared to *T. confusum,* similar to hydroprene (Arthur 2001; Arthur and Hoernemann 2004), pyriproxyfen (Arthur et al. 2009), and with methoprene applied as an aerosol (Arthur 2010, Sutton et al. 2011). Other studies with adults of these species have also shown increased survival of adults in the presence of food material (Arthur 2008a). However, when conducting studies in which immature stages are the target species, food material must be provided, which is probably a more accurate reflection of actual conditions encountered in field situations.

The methodology of exposing late stage larvae on a treated surface with food material has also been used for studies involving susceptibility of *Plodia interpunctella* (Hübner) to hydroprene (Mohandass et al. 2006) and methoprene (Jenson et al. 2009). Similarly, Wijayaratne and Fields (2010) exposed larvae of *T. castaneum* on wheat treated with methoprene in order to assess effects of the IGR on heat and cold tolerance, and also to evaluate progeny production of adults after they had been exposed as larvae on treated wheat (Wijayaratne et al. 2011). Exposing adult *Tribolium* spp. on a treated surface covered with flour, and assessing resulting progeny production, mimics a situation where a surface could be treated with an insecticide, and spillage could occur, thus creating a food patch. Female *Tribolium* spp. may be stimulated to oviposit in food patches, (Campbell and Runnion 2003), which may then absorb the insecticidal residues from a treated surface. As a result, the developing larvae would be exposed to the insecticidal residues throughout their life cycle. In our study, the rate of novaluron used for the studies reported in Table 3, where the entire life stage was allowed to develop on flour that was on a treated surface, was 1.5, 3.0, and 6.0 mg/m^2^. This method of exposure gave complete inhibition of *T. castaneum* development. In contrast, when 4-week-old larvae were exposed on the surfaces treated with 10, 20, and 30 mg/m^2^ novaluron, adult emergence occurred. These results indicated far greater effectiveness of novaluron when *T. castaneum* was exposed for their entire life cycle, and perhaps the method of evaluation used in Experiment 3 could be incorporated into new studies with IGRs.

Direct exposure procedures may be useful for evaluating IGRs, as opposed to traditional methods of dietary incorporation, because direct exposures more accurately mimic how insects would be exposed to IGRs in practical conditions. These procedures could also be used in studies with contact insecticides. The presence of food material on a treated surface leads to increases in adult survival of exposed *Tribolium* spp. (Arthur 2009), which might be somewhat offset by the suppression of progeny development offered by the insecticide. Recent studies showed that larvae and pupae of *T. castaneum* and *T. confusum* were more susceptible to pyrethrin aerosol compared to the adults (Arthur and Campbell 2008), but comparison studies of larvae versus adults have not been done with some of the newer contact insecticides that are labeled for flour beetles in the USA. Such studies would, by necessity, involve provisioning both the exposed larvae and adults so that comparisons would be valid.

Novaluron gave almost complete progeny suppression of *T. castaneum* and *T. confusum,* at application rates comparable to the IGR methoprene. Presumably, this was through inhibition of larval development. However, there are some reports of reduced fecundity after adult insects have been exposed to novaluron. Kostyukovsky and Trostanetsky (2008) exposed adult *T. castaneum* on flour treated with novaluron, transferred the adults to untreated flour, and reported reduced adult fecundity. Similar results have occurred with other insect species exposed to novaluron. Alyokhin et al. (2010) described reduced egg hatch of adult female Colorado potato beetle, *Leptinotarsa decemlineata* (Say) when fed on novaluron-treated foliage. Gökce et al. (2009) documented ovicidal effects on adult codling moth *Cydia pomonella* (L.) exposed to novaluron. As it stands, a combination of lethal and sub-lethal effects could have been responsible for reduced adult emergence of adult *T. confusum* reported in Experiment 3, because adult *T. castaneum* were allowed to feed on, and oviposit in, flour on the concrete surface treated with novaluron. Reduced fecundity has also been reported when lesser grain borer *Rhyzopertha dominica* (F.) were exposed on wheat treated with methoprene, and then transferred to untreated wheat (Daglish and Pulvarenti 1997).

In conclusion, evaluation of IGRs for control of flour beetles can be accomplished through exposure of late-stage larvae on a treated surface, or by exposing adults with food material on the treated surface and assessing progeny production. These methodologies may provide a more realistic method of assessing how the insects would be exposed in a field situation, rather than by incorporating the IGR into the diet of the insect. In addition, the promising initial results with novaluron may warrant further and more detailed evaluations for controlling stored product insects.
